# INACTIVE BEHAVIOR IN ADOLESCENT STUDENTS OF THE BRAZILIAN WESTERN AMAZON

**DOI:** 10.1590/1984-0462/;2019;37;3;00017

**Published:** 2019-06-03

**Authors:** Edson dos Santos Farias, Wellington Roberto Gomes de Carvalho, Anderson Marques de Moraes, Josivana Pontes dos Santos, Ivanice Fernandes Barcellos Gemelli, Orivaldo Florêncio de Souza

**Affiliations:** a Universidade Federal de Rondônia, Porto Velho, RO, Brazil.; b Universidade Federal do Maranhão, São Luís, MA, Brazil.; c Pontifícia Universidade Católica de Campinas, Campinas, SP, Brazil.; d Universidade Federal do Acre, Rio Branco, AC, Brazil.

**Keywords:** Motor activity, Students, School health services, Prevalence, Atividade motora, Estudantes, Saúde escolar, Prevalência

## Abstract

**Objective:**

To identify the prevalence of physical inactivity in adolescent students in the city of Porto Velho, RO, Northern Brazil, and its associated factors.

**Methods:**

School-based study, conducted with 2,694 adolescents. The self-reported variable for outcome was physical inactivity. Factors associated with inactive behavior were verified by multiple logistic regression. The independent variables were inserted into the model in hierarchical blocks.

**Results:**

The overall prevalence of inactive behavior was 39.5%. Females showed a higher prevalence of physical inactivity (46.2%) than males (31.4%). Adolescents in private schools and with reports of negative health perception had a high prevalence of physical inactivity. Regarding associated factors, the female sex showed a magnitude of association of 1.84 with physical inactivity. Being in a private school was associated with a 2.54 times greater chance of physical inactivity compared to public school students. Going to school by bus, car or motorcycle was associated with a 1.29 and 1.63 higher chance of physical inactivity respectively. Adolescents who reported having a negative health perception had 1.29 higher chance of physical inactivity, while having excess body fat showed magnitude of association of 1.36 in adolescents.

**Conclusions:**

There was a high prevalence of physical inactivity in the studied adolescents. Considering that the behavior of physical inactivity adopted during adolescence may continue in adulthood, the promotion of actions that can change this behavior may improve health in the future as well as quality of life.

## RESUMO

**Objetivo:**

Identificar a prevalência do comportamento inativo de estudantes adolescentes da cidade de Porto Velho, RO, Brasil, e os fatores associados a ele.

**Métodos:**

Estudo de base escolar realizado com 2.694 adolescentes. A variável autorreferida à exposição ao desfecho foi a inatividade física. Verificam-se os fatores associados ao comportamento inativo pela regressão logística múltipla. As variáveis independentes foram inseridas no modelo em blocos hierarquizados.

**Resultados:**

A prevalência geral de comportamento inativo foi de 39,5%. O sexo feminino mostrou maior prevalência de comportamento inativo (46,2%) do que o masculino (31,4%). Adolescentes em escolas particulares e com relatos de percepção de saúde negativa apresentaram alta prevalência de comportamento inativo. Na verificação dos fatores associados, o sexo feminino mostrou magnitude de associação de 1,84 com o comportamento inativo. Estar institucionalizado em escola particular mostrou 2,54 vezes mais chance de ter comportamento inativo em comparação aos estudantes das escolas públicas. A locomoção para escola por ônibus e carro ou motocicleta revelou magnitudes de associação com o comportamento inativo de 1,29 e 1,63, respectivamente. Adolescentes que apontaram a percepção de saúde negativa tiveram 1,29 mais chance de ter comportamento inativo em relação ao relato de saúde positiva, enquanto ter excesso de gordura corporal mostrou magnitude de associação de 1,36 em adolescentes.

**Conclusões:**

A prevalência de comportamento inativo foi alta em estudantes adolescentes. Considerando que o comportamento inativo adotado durante a adolescência pode permanecer nos anos posteriores, sugere-se a promoção de ações para a modificação de comportamento visando à melhoria da saúde e qualidade de vida.

## INTRODUCTION

Physical inactivity in adolescence is a global public health problem and is one of the main risk factors for cardiovascular and metabolic diseases.^1^ ^,^ ^2^ However, scientific temporal trends indicate that physical inactivity in adolescents is increasing in developed countries.^3^ Reports from the Brazilian Institute of Geography and Statistics (IBGE) in 2009 and 2015 revealed a reduction in the practice of physical activity, with a total of 300 minutes a week in Brazilian adolescents.^4^ ^,^ ^5^

Several studies on the variables associated with physical inactivity in adolescents have been published in several Brazilian locations,^6^ ^,^ ^7^ however, such factors identified in one locality may be irrelevant in another region. Thus, it is necessary to understand the dynamics of these factors in the regional contexts for the effective implementation of prevention and control actions.

Previous research has revealed a high prevalence of physical inacivity in adolescents in Porto Velho, RO, Brazil,^8^ ^,^ ^9^ however there is limited updated information on physical inactivity in adolescents in this city. Thus, the objective of this study was to identify the prevalence of physical inactivity in adolescent students in the city of Porto Velho, southeast of the Brazilian Amazon, and its associated factors associated.

## METHOD

A cross-sectional study performed between August and November 2015, approved by the Human Research Ethics Committee of the Federal University of Rondônia Foundation (UNIR) (Certificate for Ethical Assessment - CAAE nº 14190113.30000.5300).

The subjects of the study were adolescents between 14 and 18 years of age who were enrolled in the education network of the Porto Velho municipality. The sample size calculation was based on a 50% prevalence of physical inactivity, a sampling error of 0.02%, a 97% confidence interval and a 10% increase in the estimation for losses or refusals. Thus, the calculated sample size included 2,694 adolescents from public and private education networks. The selection process for the students was carried out in three stages using the simple randomization technique according to proportionality. There were no refusals to participate in the data collection.

The habitual practice of physical activity was obtained by the International Physical Activity Questionnaire-short version, validated for adolescents by Guedes et al.^10^ Adolescents who did not practice physical activity in the last seven days were classified as inactive.

Demographic and socioeconomic variables, sedentary behavior and health perception were self-reported. The demographic variables investigated were sex and age in years. The economic class was verified and classified according to the Brazilian Association of Research Companies (ABEP) criteria of 2014.^11^ The amount schooling of the parents in years was categorized in:

- Basic education, for parents with complete or incomplete elementary and middle school.
- Higher education, for those with complete or incomplete undergraduation or technological course.

The type of school corresponded to a private or public school.

Two hours of watching television a day was dichotomized at the cut-off point. The means of transport to go to school were also investigated: bus; car or motorcycle; and walk or cycle. The subjective perception of health was defined as health perception, combining the physical and emotional components and reflecting the perception of well-being and satisfaction with life, for example: mood, mobility, pain and discomfort, interpersonal activities, vision, sleep and energy, cognition and self-care. The answers to the question of health perception were categorized in a Likert scale with 5 options, from excellent to poor. In order to analyze the data, it was chosen to categorize positive and negative answers.

The anthropometric variables of weight, height, tricipital and subscapular skinfolds were measured. The Z score of body mass index (BMI) for age was calculated with the help of the WHO AnthroPlus program. Subsequently, the BMI for age was categorized according to the criteria proposed by the World Health Organization (WHO)^12^ as normal weight and overweight. The body fat percentage was estimated by using the equation proposed by Slaughter et al.,^13^ considering sex, race and sexual maturation.^14^ ^,^ ^15^ The procedure for categorizing fat percentage was adapted from the criteria suggested by Lohman et al.^16^ for normal and excess fat.

The Statistical Package for the Social Sciences (SPSS), version 20.0 (IBM Corp., Armonk, NY, USA) was used for statistical analysis. The prevalence and odds ratio of phyical inactivity were determined according to demographic and socioeconomic variables, sedentary behavior, health perception and body adiposity. Factors associated with physical inactivity were verified by multiple logistic regression. The independent variables were inserted in the model in hierarchical blocks. In the first block, the demographic and socioeconomic variables were inserted; in the second block, the variables of sedentary behavior; and in the third, the variables health perception and body adiposity. The final model was composed of all variables that presented a level of statistical significance lower than 0.05.

## RESULTS

Among the 2,694 adolescents investigated, the mean age was 16.24±1.06, ranging from 14.0 to 18.0 years. The overall prevalence of physica inactivity was 39.5% (n=1,064). The female sex showed a higher prevalence of physical inactivity (46.2%) in contrast to males (31.4%). Adolescents enrolled in private schools and who reported negative health perceptions showed a high prevalence of iphysical nactivity (Table 1).

**Table 1:** Prevalence (%) of physical inactivity according to demographic and socioeconomic variables, sedentary behavior, health perception and body adiposity of adolescent students.

Variables	Classification	n	Prevalence n (%)
Sex	Male	1.212	380 (31,3)
	Female	1.482	684 (46.2)
Age (years)	≤16	1.527	553 (36.2)
	>16	1.167	511 (43.8)
School network	Public	1.598	771 (48.2)
	Private	1.096	293 (26.7)
Social class	A+B	1.942	708 (36.4)
	C+D e E	752	356 (47.3)
Parents’ schooling	Basic	1.636	697 (42.6)
	Superior	1.058	367 (34.6)
Television (hours a day)	≤2	2.143	816 (38.1)
	>2	551	248 (45.0)
Transport/school	Walking or bicycle	504	191 (37.8)
	Bus	562	249 (44.3)
	Car or motorcycle	1.628	624 (38.3)
Perception of health	Positive	2.158	800 (37.1)
	Negative	536	264 (49.2)
Body fat (%)	Normal	969	354 (36.5)
	Excess fat	1.725	710 (41.1)
BMI (kg/m^2^)	Normal weight	2.042	811 (39.7)
	Overweight	652	253 (38.8)

BMI: body mass index.

Upon verification of the associated factors, the female sex showed a magnitude of association of 1.84 with physical inactivity. Students enrolled in a private school showed a 2.54 times greater chance of being physically inactive compared to public school students. The transport to school by bus and car or motorcycle revealed magnitudes of association with physical inactivity of 1.29 and 1.63, respectively. Adolescents who reported negative health perceptions had a 1.29 higher chance of physical inactivity in relation to the positive health report, while having excess body fat showed a magnitudee of association of 1.36 in adolescents (Table 2).

**Table 2:** Factors associated with physical inactivity in adolescent students.

	Classification	OR_gross_	(95%CI)	p-value	OR_adjusted_	(95%CI)	p-value
Block 1							
Sex	Male	1	(1.60-2.19)	<0.001	1	(1.56-2.16)	<0.001
	Female	1.87			1.84		
Age (years)	≤16	1	(1.17-1.60)	<0.001	-	-	-
	>16	1.37			-	-	-
School network	Public	1	(2.15-3.01)	<0.001	1	(2.13-2.98)	<0.001
	Private	2.55			2.52		
Social class	A+B	1.56	(1.32-1.85)	<0.001	-	-	-
	C, D+E	1			-	-	-
Parents’ schooling	Basic	1	(1.19-1.64)	<0.001	-	-	-
	Superior	1.39			-	-	-
Block 2							
Television (hours/day)	≤2	1	(1.10-1.60)	0.003	-	-	-
	>2	1.33			-	-	-
Transport to school	Walking or bicycle	1	(1.05-1.55)	0.013	1	(1.00-1.66)	0.046
	Bus	1.28			1.29		
	Car or motorcycle	1.3	(1.02-1.66)	0.034	1.63	(1.30-2.06)	<0.000
Block 3							
Perception of health	Positive	1	(1.36-1.99)	<0.001	1	(1.06-1.58)	0.011
	Negative	1.64			1.29		
Body fat (%)	Normal	1	(1.06-1.86)	0.021	1	(1.06-1.73)	0.017
	Excess fat	1.41			1.36		
BMI (kg/m^2^)	Normal weight	1	(0.86-1.24)	0.678	-	-	-
	Overweight	1.03			-	-	-

OR: *Odds Ratio*; 95%CI: 95% confidence interval; BMI: body mass index.

## DISCUSSION

High school adolescent students in the Porto Velho municipality have a high prevalence of physical inactivity, revealing a serious public health problem. Specifically, being female, enrolled in private school, using motorized transport to go to school, reporting negative health perceptions and having excess body fat have all been shown to be factors associated with physical inactivity.

Similarly to the results of Brazilian national reseach,^17^ ^,^ ^18^ female subjects presented a higher prevalence of physical inactivity in contrast to males. Farias Júnior et al.^19^ report that this difference between the sexes regarding physical inactivity is due to the fact that males prefer involvement in physical activities with more intensity, duration and weekly frequency. This information explains the fact that the male adolescents from Porto Velho are more likely to maintain the practice of physical activity.

Studies corroborating the present study revealed that adolescents from private schools show greater magnitude of physical inactivity when compared with adolescents in public schools.^6^ ^,^ ^7^ National research data showed approximate prevalences of physical inactivity in students in public and private schools.

In Brazil, due to private school fees, attending such schools becomes feasible for families with better economic resources.

Therefore, since the predominant characteristic of adolescents in private schools is that they come from middle or upper-class families, it is inferred that they have easy access to the technologies (i.e., watching TV, using a computer and playing video games), which cause physical inactivity. It is noteworthy to highlight that at the end of the week, when the adolescent has more leisure time (i.e., physical activity, sports and attending gymnasiums), they choose to be more sedentary than during the week.

Nowadays, walking or cycling in urban areas is difficult, mainly due to the intense traffic of cars and fear due to the increase of violence. In addition, in the Porto Velho municipality there is a long rainy season, which impedes active transport during several months of the year. Such barriers to the practice of active transport may have promoted the choice of motorized transport by bus, car or motorcycle by adolescent students. Also, a study carried out with young Filipino students^20^ revealed that the preference for motorized transport impacts on less energy expenditure.

The association between negative health perception and physical inactivity corroborates research findings in the southwest of the Brazilian Amazon with university students.^21^ In fact, by feeling unhealthy, the adolescent student may have adopted behaviors associated with physical inactivity, however caution is suggested when analyzing this association due to the possibility of reverse causality, since physical inactivity can cause malaise and diseases^22^ and, consequently, a negative health perception.

Brazilian cross-sectional studies revealed divergent results with adolescents students in the association between physical activity and body composition. Brito et al.^23^ identified a concomitant increase in physical activity and fat percentage. However, in contrast with the present study, some research did not identify an association between these variables.^24^ ^,^ ^25^ These divergences may be due to several demotivating circumstances for adolescent students with excess body fat to adopt the practice of physical activity. One circumstance would be that excess body fat can make it difficult to perform various sports or movements. In addition, adolescents with body image dissatisfaction with excess fat may be a barrier to taking up or maintaining physical activity.^26^

The present study is limited by the cross-sectional design, which made it impossible to identify the causal relationship between the independent variables and physical inactivity. We infer that the memory bias is minimal, because the collected questions are recent events. In addition, because there are differences in the methods of definition and categorization of physical inactivity, caution is recommended in the comparison between investigations. It is advised that the generalization of the results of this research is confined to the Porto Velho municipality.

An alarming prevalence of physical inactivity was evidenced in adolescent students from Porto Velho, in the southwest of the Brazilian Amazon. Female students and those enrolled in private school were more susceptible to physical inactivity. In addition, aspects such as body adiposity, inactive transport and negative health perception were also associated with physical inactivity.

Considering that physical inactivity adopted during adolescence may continue in adulthood, the promotion of actions that can change this behavior may improve health in the future as well as quality of life.
